# Bacteriological hazard analysis and critical control point (HACCP) assessment of sachet drinking water processing in some factories within Jos metropolis, Plateau State, Nigeria

**DOI:** 10.3205/dgkh000565

**Published:** 2025-07-09

**Authors:** Josephine Abbas Dayilim, Michael Macvren Dashen, Anayochukwu Chibuike Ngene, Chinedu Godspower Ohaegbu, Otumala John Egbere

**Affiliations:** 1 Department of Microbiology, Faculty of Natural Sciences, University of Jos, Jos, Nigeria; 2 Department of Microbiology, College of Natural Sciences, Michael Okpara University of Agriculture, Umudike, Nigeria

**Keywords:** sachet drinking water, bacteriological hazards, critical control points (CCPs), water quality assessment

## Abstract

**Background:**

The quality of sachet water is increasingly concerning due to the rapid growth of production industries and high consumption rates. Therefore, this study evaluated the bacteriological hazards and critical control points (CCPs) in the production of sachet water in nine factories within Jos metropolis.

**Method:**

Water samples were collected at various production stages and analyzed for total viable bacteria, coliform, and *Escherichia (E.) coli* counts. Aerial bacterial loads in each factory were also assessed. Bacterial isolates were identified using morphological, microscopic, biochemical features, and Analytical Profile Index (API) kits. Physico-chemical properties of the water products were also evaluated.

**Results:**

The finished water samples contained 1.36 to 2.2×10^6^ colony forming units (cfu)/ml, coliform 1.26 to 2.6×10^5^ cfu/ml, and *E. coli* 1.00 to 1.98×10^6^ cfu/ml, all exceeding WHO/NIS (Nigerian Standard) standards. The most prevalent bacterial isolates included *Staphylococcus aureus* (29.63%) and *E. coli* (20.37%), among others.

The physico-chemical parameters conformed to standards. However, the presence of pathogens indicated all 12 bacterial isolates as hazards.

**Conclusion:**

Seven out of ten production stages were identified as CCPs. The study concluded that all water samples from the nine factories were unfit for human consumption due to high bacteriological loads and the presence of pathogens. Stringent quality control measures, ongoing monitoring of production activities, and adherence to CCPs are recommended to meet WHO/NIS and NAFDAC (National Agency for food and Drug Administration and Control) standards.

## Zusammenfassung

**Hintergrund:**

Die Qualität von Wasser in Beuteln ist aufgrund des raschen Wachstums der herstellenden Industrie und des hohen Verbrauchs zunehmend problematisch. Deshalb sollten die bakteriologischen Gefahren und die kritischen Kontrollpunkte (CCPs) bei der Herstellung von Beutelwasser in neun Fabriken in der Metropole Jos, Plateau State, Nigeria, untersucht werden.

**Methode:**

Wasserproben wurden in verschiedenen Produktionsstadien entnommen und auf die Gesamtzahl kultivierbarer Bakterien, auf Coliforme und *Escherichia (E.) coli* untersucht. Die bakterielle Belastung der Luft in jeder Fabrik wurde ebenfalls untersucht. Die Bakterienisolate wurden anhand von morphologischen, mikroskopischen und biochemischen Merkmalen sowie mit Hilfe von API-Kits identifiziert. Ferner wurden die physikalisch-chemischen Eigenschaften der Wasserprodukte bewertet.

**Ergebnisse:**

Die fertigen Wasserproben enthielten zwischen 1,36 bis 2,2×10^6^ koloniebildende Einheiten (KbE)/ml, davon Coliforme zwischen 1,26 und 2,6×10^5^ KbE/ml und *E. coli* zwischen 1,00 und 1,98×10^6^ KbE/ml. Damit waren alle Werte über dem WHO/NIS-Standard. Zu den am häufigsten vorkommenden Bakterienisolaten gehörten unter anderem *Staphylococcus (S.) aureus* (29,63%) und *E. coli* (20,37%).

Die physikalisch-chemischen Parameter entsprachen den Normen. Das Vorhandensein von Krankheitserregern wies alle 12 bakteriellen Isolate als gefährlich aus. Sieben von zehn Produktionsstufen wurden als CCPs identifiziert.

**Schlussfolgerung:**

Die Studie ergab, dass alle Wasserproben aus den neun Fabriken aufgrund der hohen bakteriellen Belastung und des Vorhandenseins von Krankheitserregern für den menschlichen Verzehr ungeeignet waren. Strenge Qualitätskontrollmaßnahmen, eine laufende Überwachung der Produktionsaktivitäten und die Einhaltung der CCPs werden empfohlen, um die Standards der WHO/NIS und der NAFDAC (National Agency for Food and Drug Administration and Control) zu erfüllen.

## Introduction

The consumption of sachet drinking water in Nigeria has been on the rise, irrespective of the NAFDAC (National Agency for Food and Drug Administration and Control) certification [1]. The challenges associated with drinking water have spurred the production of packaged drinking water by private enterprises, many of which have little knowledge of good manufacturing practices (GMP) [2]. Despite NAFDAC’s strong efforts in regulating and ensuring the quality of sachet water, compliance among manufacturers remains low. This has led to a growing number of public health issues, including typhoid, diarrhea, and other waterborne diseases linked to the consumption of sachet water [3].

The integrity of sachet water is often questionable, with reports indicating that many vendors do not treat their water before selling it to the public. Furthermore, numerous producers fail to adhere to the standards set by NAFDAC and the World Health Organization (WHO) [4]. The increasing consumption of sachet water in Nigeria has raised concerns about its source, quality, and potential health consequences. Contamination can occur during various stages, e.g., processing, transportation, and improper handling by hawkers, as well as from the water sources used in production. Contaminated drinking water not only poses health risks but also has significant socio-economic and political implications [5].

Studies on the bacteriological quality of sachet water in African cities have documented various levels of contamination [6], [7]. Many brands fall below WHO standards for drinking water, calling their quality into question. Adherence to production and analytical standards is often inadequate, with many factories lacking the appropriate technology to achieve these standards. While some manufacturers use advanced techniques such as ozonization and reverse osmosis, others rely on basic methods like boiling well-water and using unsterilized filtration materials [8].

Waterborne diseases result in over 5 million deaths globally each year, with more than 50% linked to microbial intestinal infections such as cholera and typhoid [9]. Contaminated water often harbors pathogens like *Salmonella spp*., *Shigella spp*., *Escherichia (E.) coli*, and *Vibrio cholera*, as well as protozoan agents such as *Entamoeba* *histolytica*, *Giardia* *lamblia*, and *Balantidium* *coli* [10]. The most common contaminants of raw water sources are human sewage and fecal pathogens. In 2006, the CDC reported that waterborne diseases caused approximately 1.8 million deaths annually, with 1.1 billion people lacking access to proper drinking water [11]. Improving access to safe drinking water is crucial, as it can lead to significant health benefits. Drinking water must be free from harmful contaminants and possess acceptable physical, chemical, and organoleptic properties [12].

In Nigeria, access to potable water is a major issue, with many people relying on surface water, hand-dug wells, rainwater, and boreholes [13], [14]. The quality of piped water is deteriorating due to inadequate treatment plants, untreated sewage discharge, and inefficient water distribution systems [15]. In developing countries like Nigeria, the lack of information on pathogenic organisms in drinking water creates uncertainties about its quality. Studies have found sachet water contaminated with bacteria such as *Bacillus* spp., *Pseudomonas* spp., *Klebsiella* spp., and *Streptococcus* spp., as well as *Cryptosporidium* *oocysts* [16]. 

The overall quality of sachet water depends on the source water, type of sachet water produced, and production location [5]. Previous investigations have shown that market-sold water is often of poor microbiological quality, with factory-bagged and hand-filled polythene-bagged water also failing to meet standards [17], [18]. These studies identified the presence of heterotrophic bacteria, fecal coliforms, and other contaminants. Surveillance by the National Agency for Food and Drug Administration and Control (NAFDAC) revealed widespread non-compliance with quality standards, prompting the agency to issue guidelines for the production of packaged water [19]. However, compliance with these guidelines remains problematic. 

This study aims to assess the bacteriological hazards and critical control points (CCPs) in sachet-water production factories within Jos metropolis, Nigeria. The study will determine bacterial loads at various production stages, isolate and identify bacterial contaminants, and analyze the physico-chemical properties of source and finished water. The findings will help design a decision tree to identify CCPs and propose stringent quality control measures to ensure compliance with WHO/NIS and NAFDAC standards. 

## Materials and methods

### Study area

**Figure 1:**
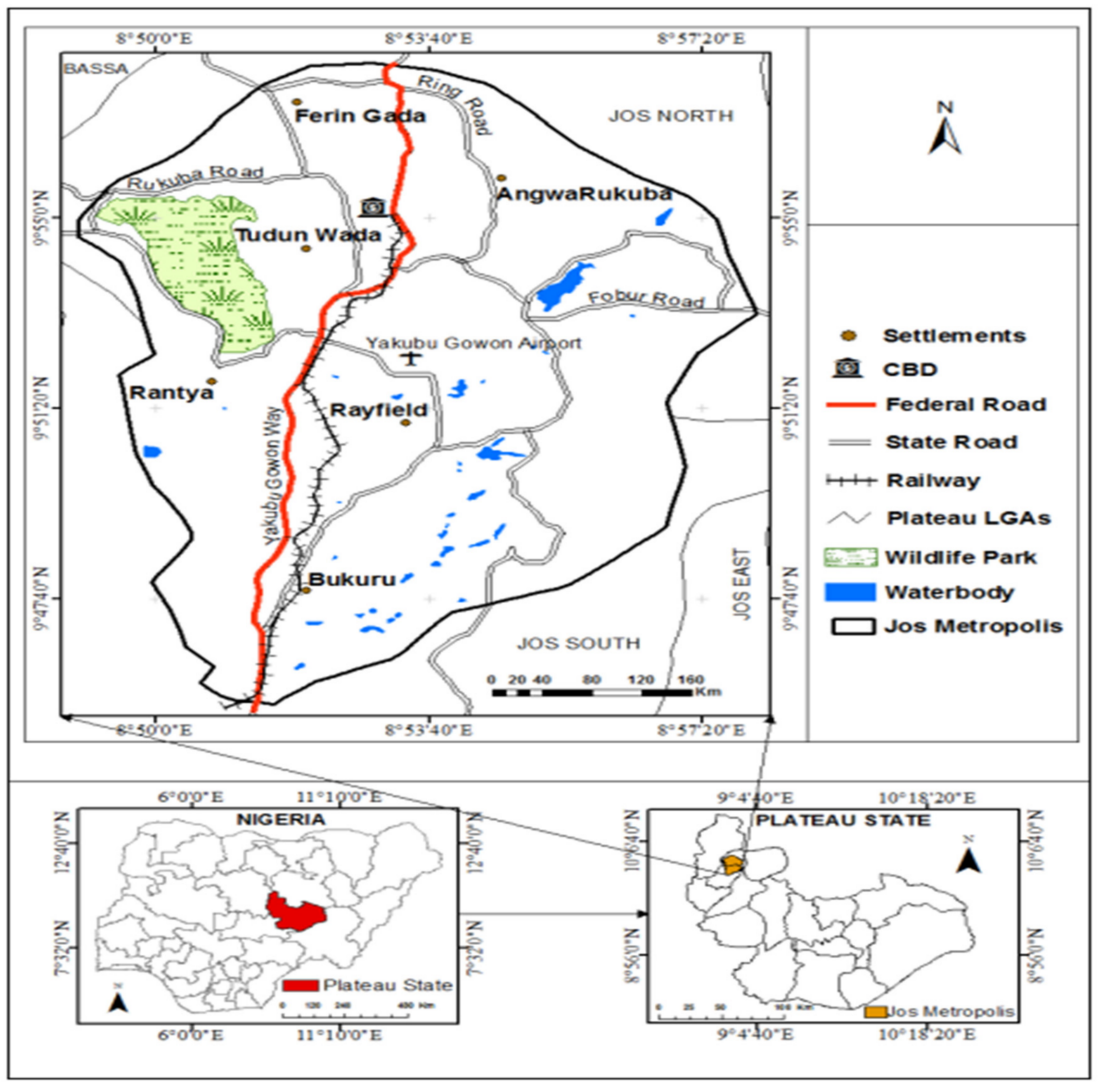
Map of Jos metropolis showing the study area

The study was conducted in Jos metropolis, the administrative headquarters of the Plateau State in Nigeria. The study area consists of parts of Jos North and Jos South Local Government Areas with populations of 429,300 and 306,716, respectively, as estimated in the 2006 National Census (Figure 1 (Fig. 1)). The metropolitan area covers about 319.7 km² and situated at a relatively high altitude of 1,217 meters (3,993 feet) above the level. The climate is relatively mild compared to other parts of Nigeria, with Jos enjoying a tropical rainy climate with distinct wet and dry seasons. The average of autumn rainfall is about 1,250 mm, and the maximum monthly rainfall is 200 mm in July and August. The mean temperature is about 22°C for the entire year, while the monthly temperature ranges between 19°C in December to 25°C in April. Jos is the largest town in Plateau State and its early development was associated with the exploitation of tin on the Jos Plateau and the building of railway lines linking the town with Port Harcourt and Lagos. This development connected the area to the global economy, thereby attracting migrants such as the Hausas, Igbos, Yorubas from different parts of Nigeria, and even Europeans, who make up over fifty percent of the town’s population. Thus, Jos is one of the most cosmopolitan cities in Nigeria.

### Experimental design

From each brand of sachet water, a bag containing 20 sachets was purchased. Half (ten sachets) were stored at ambient temperature, while the other half (ten sachets) were refrigerated. Samples collected were subjected to microbiological and physicochemical analysis; the latter included color, taste, odor, turbidity, temperature, pH, electrical conductivity, total dissolved solids, total hardness, chloride, sulphate, nitrate and phosphate. 

### Sample collection

One bag (containing 20 sachets) of sachet water was collected from 9 randomly selected sachet-water factories within Jos metropolis from CCPs identified by the researcher.

### Culture media

MacConkey broth (Titan Biotech), Nutrient agar (Titan biotech) and Mannitol Salt Agar (Oxoid) were used for isolation of bacteria.

A conical flask was filled with the recommended amount of each culture medium, with appropriate amount of distilled water added according to manufacturer’s instruction. The agar was entirely dissolved in the suspension by boiling it. All of the media were then autoclaved for 15 minutes at 121°C and 15 pounds of pressure to sterilize them. Following sterilization, sterile petri dishes were aseptically filled with 15 ml of each medium, which was then left to solidify. The petri dishes were labeled accordingly.

### Total viable count

The method used for enumeration of bacteria was the multiple tube fermentation technique as described previously [20]. A presumptive test was done using MacConkey broth. Using a 10-ml sterile disposable syringe, 10 ml of the sample was withdrawn and dispensed aseptically into five tubes containing 10 ml of MacConkey broth double strength, then 50 ml of the sample was dispensed into one tube containing 50 ml of MacConkey broth double strength. Each bottle test tube contained an inverted Durham tube. The test tubes were closed tightly and shaken to distribute the sample uniformly throughout the medium and to make sure the inverted Durham tube was full of broth, with no air bubbles trapped inside it. The test tubes were incubated at 37°C for 24 hours. After 24 hours, the tubes from the presumptive fermentation test showing gas and acid formation were recorded and the corresponding Most Probable Number (MPN) index was determined from the probability table. Those positive tubes were streaked onto nutrient agar plates, from which distinct colonies were removed and subcultured in nutrient agar slant tubes, labeled appropriately and refrigerated for further assay. 

### Identification of bacterial isolates

Gram staining was done to differentiate gram-positive samples from gram-negative samples. The methodology described previously was employed [21]. Isolates were identified based on gram-staining and biochemical tests using the API (Analytical Profile Index) 20E test. 

### Coliform and E. coli

Multiple tube fermentation tests were used to count the total coliform bacteria and *E. coli* (APHA, 2005[22]). The Most Probable Number (MPN) three-tube assay method was used to determine the coliform count. MacConkey broth was used for the presumed coliform test. Ten milliliters of sterile Double Strength Lactose Broth (DSLB) was pipetted into the first three sets of tubes, whereas Single Strength Lactose Broth (SSLB) was employed in the second and third sets. Before they were sterilized, every tube contained a Durham tube. Using sterile pipettes, 10-, 1-, and 0.1-ml water samples were added to each of the three sets of tubes. The tubes were incubated for 24–48 hours at 37°C to estimate total coliform bacteria and for 24–48 hours at 44.50°C to estimate *E. coli*; they were subsequently checked for the formation of gas and acid. Acid production was indicated by color change of the broth from reddish purple to yellow, and gas production was indicated by entrapment of gas in the Durham tube. The MPN was then determined from the MPN table for the three sets of tubes [23]. 

To confirm coliforms, Durham tubes were used to transfer a loopful of culture from a positive tube from the presumptive test into a tube of Brilliant Green Lactose Bile (BGLB) broth for the confirmation test. For 24 to 48 hours, the tubes were incubated at 37°C for total coliform bacteria and 44°C for *E. coli*, and gas generation was monitored [24]. 

A loopful of broth from a positive tube was streaked onto an Eosin Methylene Blue (EMB) agar plate for pure colonies to complete the test. For 24 to 48 hours, the plates were incubated at 37°C. *E. coli* colonies were further identified growing on EMB agar. It was determined that colonies with a metallic green sheen were rod-shaped *E. coli* coliform bacteria [24]. 

### Bacteriological hazard analysis and critical control points (HACCP) of sachet drinking water processing in factories

#### Principle 1 – hazard identification and analysis

**Figure 2:**
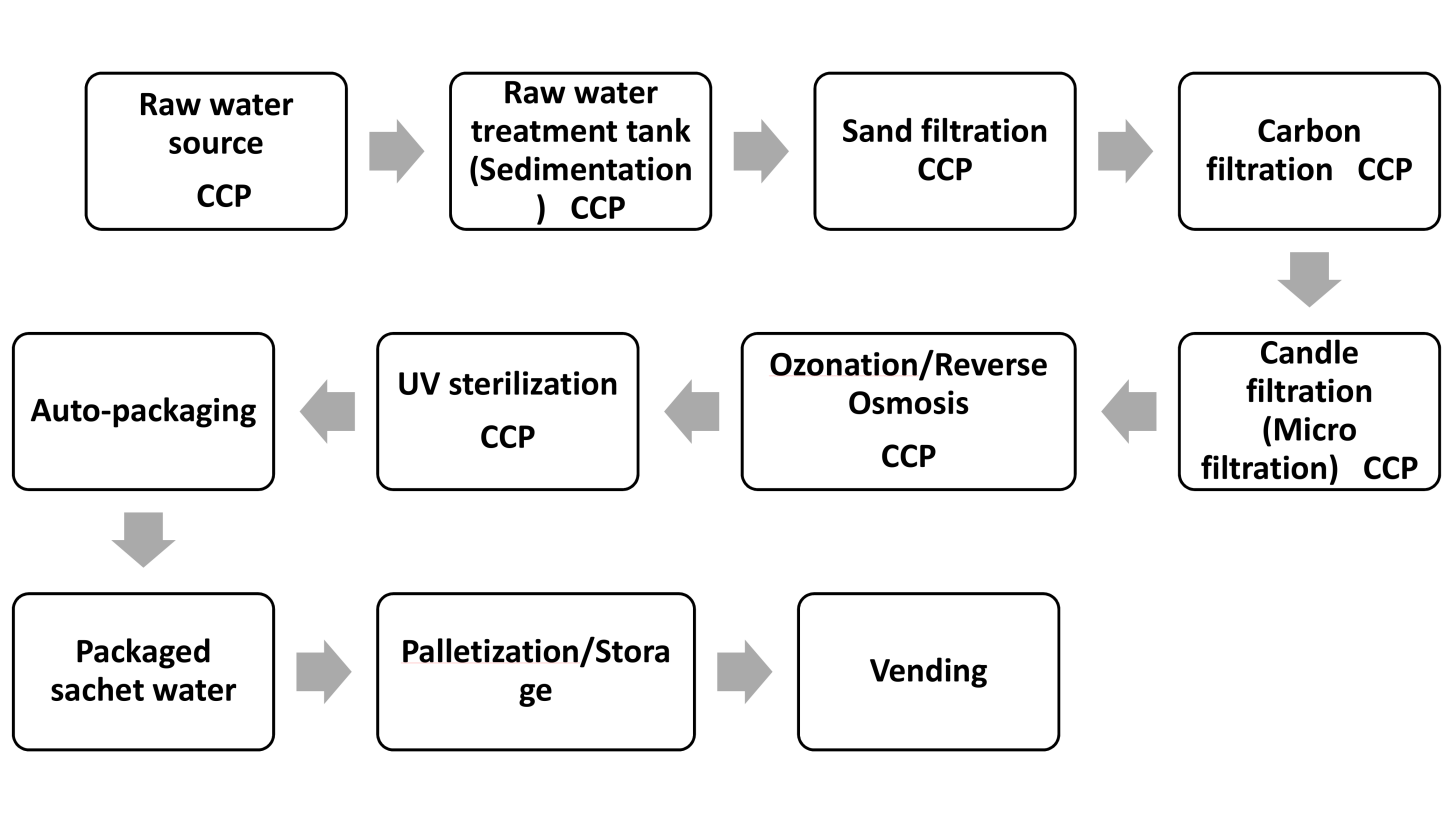
Decision tree for determining Critical Control Points (CCPs)

Hazard identification involves determining potential biological, chemical or physical contaminants that can enter the food product, causing adverse health effects [25]. The researchers held a brainstorming session with the project supervisors to identify potential hazards and their relative risks. (Fig. 2)

#### Principle 2 – determining critical control points CCPs

Following the hazard and risk analysis, the researchers determined CCPs. Each step in the production process identifies a point at which appropriate control measures can be applied to mitigate the risks of microbial contamination. To determine whether these were indeed critical points, the researchers used the US Food and Drugs Administration decision tree [26], as shown in Figure 2 (Fig. 2).

#### Decision tree for determining CCPs of a selected water-producing factory

For all process steps (source water, raw water/treatment tanks, treatment plant, water packaging machine and packaged sachet water). The decision of “yes” or “no” CCP for microbial hazard had to be made (Table 1 (Tab. 1)). 

### Physico-chemical properties

#### Taste and odor

Small volumes of each sample were tasted with the tongue and then immediately rinsed off the tongue with taste-free distilled water. Twenty milliliter (20 mL) of each water sample was poured into a clean beaker, then shaken vigorously and brought close to the nose to test for any odor present. The result was recorded accordingly as proposed previously [27], [28]. 

#### Turbidity

10 ml of deionized water was poured into a cuvette which was used to standardize the turbidity machine. Then, 10 mL of each sample was poured into cuvettes, inserted into the machine, read and recorded at 430 nm on a D70 Jackson turbidity meter as described elsewhere [22].

#### Temperature, pH, total dissolved solid (TDS) and electrical conductivity

A 40 mL volume of each sample was poured into a beaker. The rod of the Hanna machine (HI 98129, HANNA) was then inserted into the beaker containing the sample, the machine was turned on, and the reading for each of the parameter was noted and recorded. 

#### Total hardness

25 ml of the water sample and 25 ml of distilled water were transferred into a 250-ml conical flask, 2 ml of buffer solution and 0.1g of Eriochrome Black T dye were added and titrated against ethylenediamine tetra acetic acid (EDTA) as described elsewhere [22].

#### Chloride

100 ml of the water sample was transferred into a 250-ml conical flask, two to three drops of potassium chromate was added, and the content was swirled for a few minutes. The solution was then titrated against silver nitrate solution until a dirty reddish precipitate was obtained [22]. 

#### Sulphate

25 ml of the water sample and 25 ml of distilled water were transferred into a 250-ml conical flask. One gram of barium chloride (BaCl) was added, stirred and allowed to stand for 30 minutes. The color intensity was then measured at 430 nm on a Sherwood 175 colorimeter [22]. 

#### Nitrate

A clean, dry crucible was filled with a 100-ml sample of water, then evaporated in an oven set at 100°C until completely dried. It was then taken out and let cool before 2 ml of phenol disulphonic acid was added and uniformly swirled for 10 minutes. After that, 10 ml of distilled water was added, followed by 5 ml of an ammonia solution. On the Sherwood 175 colorimeter, color change was detected at 430 nm [22]. 

#### Phosphate

100 ml of the sample was transferred into a 250-ml conical flask, 1 ml of ammonium molybdate reagent and 1 drop of stannous chloride were added and allowed to stand for 12 minutes. Color change was read at 600 nm in the same colorimeter [22]. 

### Sachet-water production process

**Figure 3:**
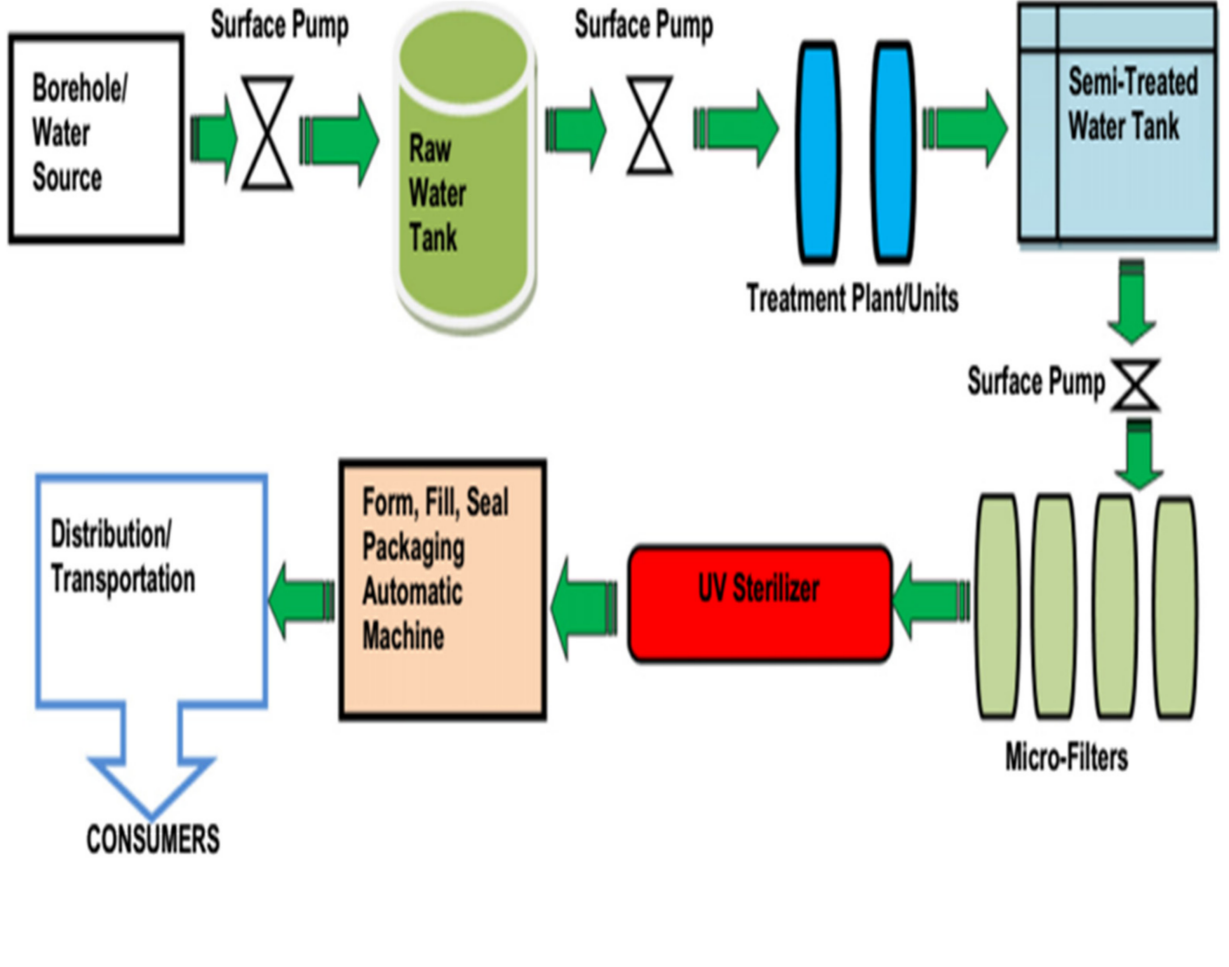
Flow diagram of sachet-water production process

With the aid of a 1-HP submersible pump, water from the source (borehole or public mains) is pumped into a tank containing raw water, where flocculation, aeration and sedimentation take place. From the raw-water tank, water is pumped using 1-HP pump through an industrial-grade sand filter and an activated-carbon filter into a PVC tank. This semi-treated water is pumped through a reverse osmosis (RO) system, if available, then through microfilters of different pore sizes (5 µ, 2 µ,1.0 activated carbon and 0.5 µ microfilters) to a UV water sterilizer for final sterilization. The treated water is then packaged using an auto-filling and -sealing machine into low density polythene sachets. It is then distributed for human consumption (Figure 3 (Fig. 3)).

### Statistical analysis

ANOVA was utilized to examine the results about the number of microorganisms and physicochemical properties at varying CCPs. The LSD (Least Significant Difference) test was used to separate the means given a significant difference between the treatments. 

## Results

### Bacterial loads (total viable count, coliform bacteria and E. coli counts) at various stages of sachet water production in selected factories

The total viable counts (TVC) at various stages of sachet-water production in selected factories in the Jos metropolis is presented in Table 2 (Tab. 2). The ambient air in the factory had the highest mean bacterial load of 3.279x10^6^ cfu/mL, followed by raw water, which had a mean bacterial load of 3.121x10^6^ cfu/mL. The least load was found for the water that had been microfiltered, which had a mean bacterial load of 1.415x10^6^ cfu/mL. 

Table 3 (Tab. 3) presents the total *E. coli* counts (TECC) taken at various stages of sachet-water production in selected factories. Raw water had the highest mean *E. coli* count of 2.97x10^5^ cfu/mL, followed by sand-filtered water with 1.933x10^5^ cfu/mL. The least load was found after UV exposure, with 1.177x10^5^ cfu/mL.

Table 4 (Tab. 4) shows the total coliform counts (TCC) at various stages of sachet-water production in selected factories. Raw water had the highest mean total coliform count of 2.966x10^6^ cfu/mL, followed by sand-filtered water with 1.54x10^6^ cfu/mL; the least load was found for micro-filtered samples, with 0.740x10^6^ cfu/mL. 

### Identified critical control points (CCPs) in the production line

Results from distributed questionnaires in the factories revealed that microbial contamination was the greatest risk at a number of process steps posing a hazard. The flow diagram was used to aid this process (Table 5 (Tab. 5)) and it was established that the most important risk in sachet water production in the factories was microbial contamination related to the location of the boreholes. A physical inspection of the water factories further confirmed these process steps as potential areas of microbial contamination. 

The critical control points (CCPs) determined using the decision tree method is as presented in Table 5 (Tab. 5). For question 1, majority said yes that control measures exist for all listed critical control points. For question 2, majority of the respondents said No for source water (88.9%) and treatment plant (91.1%) while for other critical control points majority said yes. For question 3, majority said yes for source water and treatment plant critical control points while other critical control points had zero respondents. For question 4, majority said No for source water and treatment plant critical control points while other critical control points had zero respondents. Finally, all respondents said yes that source water, raw water/treatment tanks, treatment plant, Water packaging machine and Packaged sachet water were all critical control points in a sachet water producing factory. 

### Bacterial contaminants at various stages of sachet-water production in the factories

Table 6 (Tab. 6) presents the morphological, microscopic and biochemical observation of the different isolates from factory environment and water samples. The isolated organisms using the API 20E test were *E. coli*, *S. aureus, Shigella (S.) dysentariae*, *Klebsiella (K.) pneumoniae*, *Proteus (P.) mirabilis*, *Citrobacter* spp., *Pasteurella* spp., *Acinetobacter* spp., *Pseudomonas* spp., *Enterobacter* *(E.) aerogenes*, and *Salmonella* *Typhi*.

*S. aureus* occurred in all nine (9) factories, followed by *E. coli*, while the least frequent bacterium was *Pseudomonas (P.) luteola* (Table 7 (Tab. 7)). Also, *S. aureus* had the highest occurrence (29.6%), followed by *E. coli* with 20.37%, *S. dysenteriae* with 11.1%, and finally *P. luteola* (1.9%). The factory with the highest bacterial occurrence was factory “d” with 15.0%, followed by factory “a” with 13.3%, while factory “” had the least, with 6.7% (Table 8 (Tab. 8)). 

### Physicochemical properties of source water and finished water

Clear differences existed between the factories. For instance, in raw water, the highest pH was 7.2, the lowest 6.0, in treated water 7.1 and 6.0. resp. The highest turbidity in raw water was 5.52 and the lowest 0.19; in treated water 4.85 and 0.14, resp. The highest chloride level in raw water was 250 mg/L, and the lowest was 160 mg/L; in treated water, the highest and lowest values were 100 mg/L and 87 mg/L, resp. The highest nitrate level in raw water was 5.2 mg/L, the lowest was 1.5 mg/L; in treated water, these were 0.02 mg/L and 0.01 mg/L, resp. (Table 9 (Tab. 9) and Table 10 (Tab. 10)). 

### Decision tree for critical control points

In a decision tree, every internal node represents a “test” for an attribute (such as whether a coin flip heads or tails), every branch represents the test’s result, and every leaf node represents a class label (the choice made after calculating all attributes). The structure resembles a flowchart. It is a kind of flowchart that breaks down the various options for action, making decision-making easier.

To ascertain if a step in a production process is a CCP or not, the decision tree asks four questions. Decision-tree methods can be broadly classified into four categories: identification, reduction in variance, chi-square, and CART (classification and regression trees). Using decision trees, items can be classified based on their learning objects and solutions to classification problems can be found. They can also be applied to regression issues or to forecast continuous results from unanticipated data. Classification trees are tree models in which the target variable can take a discrete set of values; leaves in these trees indicate class labels, and branches indicate the combination of features that lead to those class labels. Regression trees are decision trees in which the objective can accept continuous data, usually real numbers. A decision tree can be used in decision analysis to formally and visually reflect decisions and decision-making. This research analyzed water quality data from several factories by presenting a decision tree-based categorization strategy. Table 1 (Tab. 1) shows a simplified result of answers to the 4 fundamental questions in determining CCPs. Figure 2 (Fig. 2) shows a decision tree for locating critical control points in a factory that produces sachet water. 

## Discussion

The physical and bacteriological state of sachet water is a very important aspect that should be observed by all the sachet-water factories. Sachet water is generally of good quality for drinking, but if it is not properly protected during packaging and transit, it could become contaminated [5]. The study findings showed that most of the sachet-water samples had total coliform bacteria counts above the acceptable levels for human consumption. These findings are largely in agreement with the findings of other studies [29], [30], [31]. Moreover, the present findings resemble those from a study carried out in Lahore, Pakistan [32], where 4 out of the 15 sachet-water brands tested did not meet WHO and national standards of 0 cfu per 100 ml. Another study in Nigeria [33] reported that sachet-water samples contained coliform bacteria. The rate of contamination varied according to the type of packaged water. This finding agrees with studies conducted previously [29], [34]. Furthermore, none of the samples of sachet water were positive for fecal coliform. This finding is consistent with those of other studies, in which no microbial indicators of fecal contamination in packaged water were found [30], [35]. Otherwise, in Ghana, three studies reported contamination of sachet water with coliforms [36], [37], [38]. The coliform contamination was only found at the point of production (raw water) and therefore raises issues of hygiene at these places. These contaminated samples might have contaminated an entire batch of sachet water produced that day, and therefore could affect a larger proportion of the public. This contamination of water with bacterial pathogens could be attributed to poor hygiene practices at the factories. Also, this may be due to the inability of the filtration systems to effectively remove all bacterial pathogens from the raw untreated water. Defects in the disinfection process, due to insufficient chlorine added or a faulty UV system, may also foster the occurrence of bacterial pathogens in sachet water. 

The WHO [11] sets a pH standard of 6.5–8.5 for drinking water. In one factory for treated water, the pH was <6.5. Values below 6.5 makes water too acidic for human consumption, as it can cause serious health complications due to acidosis. None of the factories investigated here had a pH value below the recommend standard (6.5). This factory compliance rate is higher than the percentage found in research done on water samples from Accra, Odumase-Krobo, and Nsawam by Obiri-Danso et al [39]. They reported a 78% compliance rate for pH. Thus, it seemed unlikely that the sachet water sold in the research region would result in any pH-related health issues, such as acidosis or alkalosis [40]. In general, the pH scale can be used to determine the hardness or softness of water. Pure water has a pH of 7. Water is generally classified as basic if its pH is >7 and acidic if it is <7. Surface water systems typically have a pH between 6.5 and 8.5, while groundwater systems typically have a pH between 6 and 8.5 [41].

Regarding the increase in pH from raw water to final products, the findings of this study suggest that an increase in the bacterial population is responsible for the production of basic metabolic waste products. A distinct, flat, and disagreeable taste and the production of scale-like deposits are the results of excess alkalinity. The total compliance of sachet-water samples in this study contradicts observations by Aremu et al [42], who reported low total dissolved solid (TDS) contents and a high pH in their study. Studies conducted in various places in Ghana all recorded compliance with physical and chemical quality standards [36], [37], [43]. The pH range of 6.8 to 7.1 recorded here could be due to the filtration of raw untreated water before packaging; this treatment process has the potential to reduce the concentration of TDS.

There were elevated nitrate concentrations in water samples at the production point, which is the raw water sample (1.5–5.2 mg/L), and low concentrations at the retail point (0.01–0.02 mg/L), far lower than the standard of 3 mg/L. This result is similar to results obtained a previous study [39] in Accra. However, Addo et al [44] reported that 25% of sachet-water samples in Nigeria had elevated nitrate concentrations. The low nitrate concentration in the samples could be attributed to a very low concentration of the compound in the raw untreated water, which puts less stress on the treatment facilities, thus promoting a further reduction in concentration. Elevated nitrate concentrations in drinking water can lead to methemoglobinemia, a condition in which nitrate binds to hemoglobin, reducing the blood’s ability to carry oxygen. This condition is more common in infants than adults because of the low stomach-acid content of infants. 

As per the national and WHO standards, sachet water should have turbidity of less than 5 NTU and a pH range of 6.5–8.5. The physical properties of packaged water showed that pH and turbidity were within the permissible limits and thus suitable for consumption. These results are similar to studies conducted by other authors [34], [45], [46]. However, these results contradict results from studies carried out by Sheshe et al [47] and Cheabu et al [48], which showed pH values for sachet water ranging from 5.4 to 7.6, as well as Ibrahim et al [49], who reported a pH range from 5.3 to 5.5, which implies that some of the sachet water is not suitable for human consumption. Yet other authors [50] showed that sachet water had pH and turbidity values outside the acceptable range. 

The sachet water analyzed in this study had a storage temperature range of 25.0 to 25.4°C, which is within the WHO/NIS standard value for quality water [7]. This could be due to low temperature of 18 to 28°C in Jos, Plateau State, during the period of this study. However, these temperatures fell within the optimal growth temperature (20–45°C) for mesophilic bacteria [51]. This result is like that found by other authors [52], who reported 24.94°C and 24.81°C, respectively, for average temperatures of plastic sachet and bottled water brands. Danso-Boateng et al [53] state that temperatures in this range are ideal for mesophilic bacteria (which include agents that cause disease in humans) to thrive to their optimum potential. Over time, this behavior may encourage the growth of an unwanted taste and odor in water [53]. Nonetheless, 2013 State Water Quality Control Board research found that higher temperatures between 29 and 32°C limit the survival period of parasitic worm cysts and ova, such as *Schistosoma* ova [54].

According to the WHO [7], the electrical conductivity measurements for the sachet water under investigation fell between 0 and 1,000 µs/cm, which is the range of the standard conductivity for clean water. Electrical conductivity is determined by the number of ions in the water [55]. These conductive ions are produced by inorganic substances such carbonate compounds, alkalis, chlorides, sulfides, and dissolved salts [52].

Another term for substances that dissolve into ions is electrolytes. The conductivity of water is directly proportional to the concentration of ions (more ions=higher conductivity; fewer ion=lower conductivity) [53]. The sachet-water samples tested here exhibited low conductivity, could be the result of fewer dissolved ions or salts in the sachet water. The WHO/NIS (0–5 NTU) standard for turbidity was met by the treated sachet water under investigation [7]. The reason for this could be that, in contrast to the raw water, sachet water undergoes a series of filters or effective filter media during production to eliminate suspended solids, trace elements, and suspended clay particles [52].

One possible explanation for the greater probability of sachet water being contaminated could be the unhygienic practices observed in sachet-water factories. The most typical water source in Nigeria for the manufacture of sachet water is a borehole, which is more likely to be contaminated due to the sites of construction [30]. Sachet water may also possibly be contaminated before packaging, as observed in a different study [56]. Other reasons could be leather sachets that can easily be punctured. Additionally, sachet water is most likely to be hand filled with water of suspect quality under unhygienic conditions and sold cheaply. 

In this study, it was possible to identify CCPs using the HACCP method, showing that the greatest hazards were microbial contamination of source water, cases of recontamination, regrowth of thermotolerant coliforms along process steps, and in some cases, failure of treatment equipment to remove the same. Source water had the highest failure rate in all process steps, followed by the raw-water tanks, primary filtration units and water from ultraviolet/micro-filtration treatment, as well as the finished product (packaged sachet water). These findings suggest the need for the government and other stakeholders to intensify surveillance activities and enforce strict hygienic measures for packaged water industries to improve water quality. 

The majority of sachet-water samples were contaminated with total coliform bacteria above acceptable limits for human consumption. High total bacterial counts were recorded in raw-water sources of sachet-water-producing factories. The presence of total coliform in sachet water is linked to contamination and improper storage. High bacterial counts in raw water indicate that the source water is contaminated and not fit for consumption before treatment. Bacterial pathogens isolated and identified were Escherichia spp., *Staphylococcous* spp., *Shigella* spp., *Klebsiella* spp., *Proteus* spp., *Citrobacter* spp., *Vibrio* spp., *Pasteurella* spp., *Acinetobacter* spp., *Pseudomonas* spp., *Enterobacter* spp., and *Salmonella* spp. This implies that some sachet water brands act as carriers of bacterial pathogens. 

The physical properties of packaged water investigated showed that pH (with one exception) and turbidity were within the permissible limits, which would indicate that sachet water is suitable for consumption. There were elevated nitrate concentrations in raw water samples and low nitrate concentrations in the finished products. The temperature range was between 25.0 to 25.4°C, which complies with the WHO standard for quality water.

The most prominent hazard recorded was microbial contamination of source water, cases of recontamination, regrowth of thermotolerant coliforms along process steps, and failure of treatment equipment to remove contaminants. Source water had the highest failure rate in all process steps followed by the raw water tanks, primary filtration units and water from the ultraviolet/micro-filtration treatment, as well as the finished product.

## Conclusion

The producers of sachet water should be made aware of the need to keep their premises clean to prevent contamination. The source, treatment process, and storage facilities of packaged water factories should be investigated. The findings suggest need for the government and other stakeholders to intensify surveillance and monitoring activities and enforce strict hygienic measures in this rapidly expanding industry to improve water quality. There is need for good practice in the distribution and storage of sachet water if quality problems are to be eliminated. NAFDAC should stipulate a requirement for all factories to comply with the principles of HACCP and report the results. This would ensure the use of HACCP across each sachet factory and lead to overall improvement in sachet water product quality. Adopting HACCP in sachet-water production will greatly improve consumer confidence in sachet water, which is has ebbed due to the perception that hygiene standards are not always maintained during sachet-water processing. These measures, if implemented, would undoubtedly improve the quality of sachet drinking water. 

## Notes

### Authors’ ORCIDs

- Dashen MM: https://orcid.org/0009-0007-9587-0314
- Ngene AC: https://orcid.org/0000-0003-4730-2834
- Egbere OJ: https://orcid.org/0009-0001-6908-763X

### Funding

None. 

### Competing interests

The authors declare that they have no competing interests.

## Figures and Tables

**Table 1:**
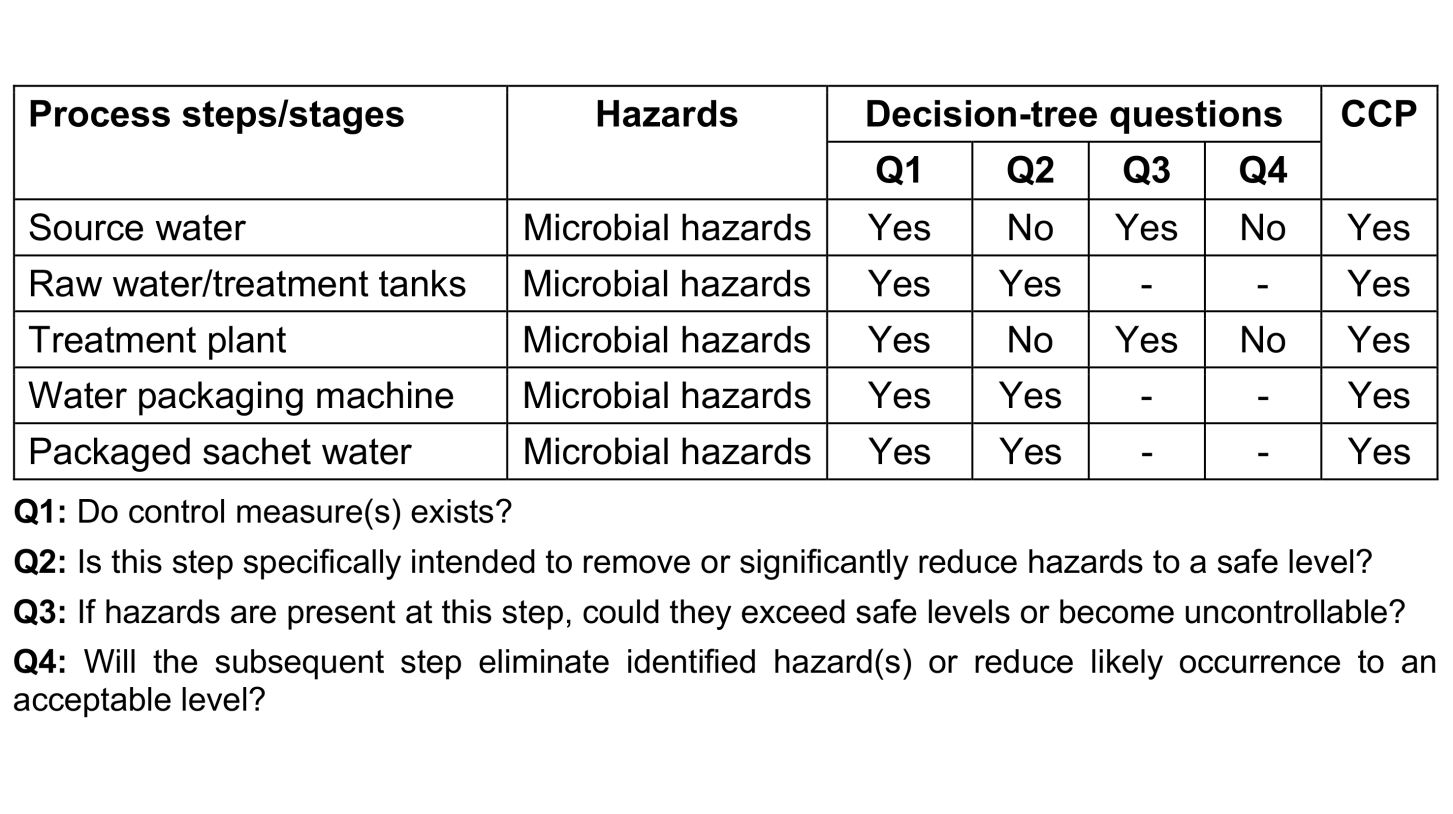
Determination of critical control points (CCPs)

**Table 2:**
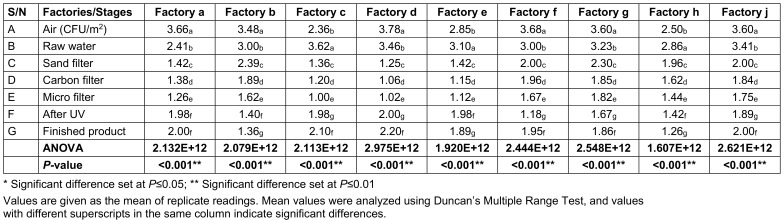
Total viable counts (TVC) of bacterial loads of factories environment and water samples taken at various stages of production (CFUx10^6^)

**Table 3:**
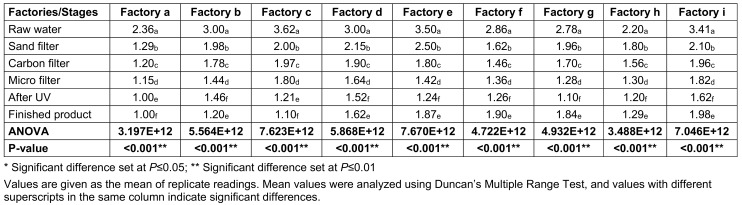
Total *E. coli* counts (TECC) from the environment of various factories and water samples taken at different stages of production (CFUx10^5^)

**Table 4:**
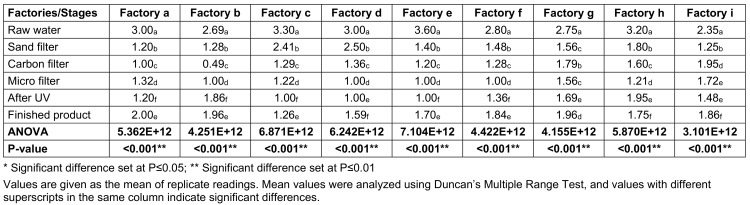
Total coliform counts (TCC) from the environment of various factories and water samples taken at various stages of production (CFUx10^6^)

**Table 5:**
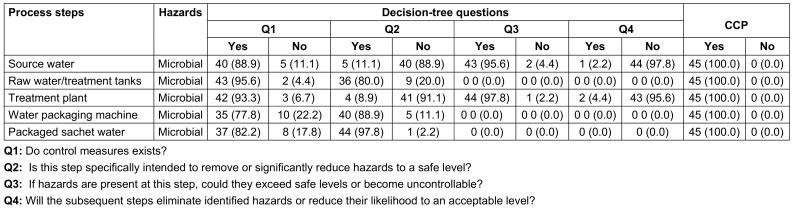
Critical control points (CCPs) determined using the decision tree method

**Table 6:**
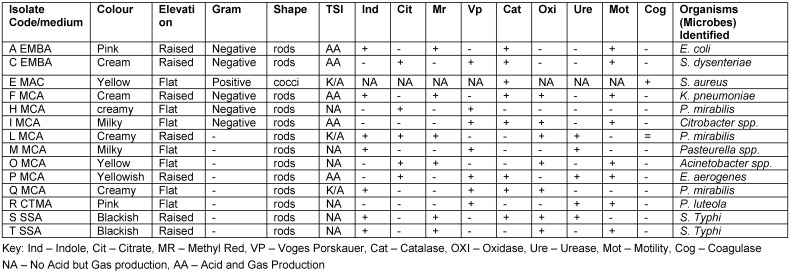
Morphological and biochemical characteristics of the different isolates from factory environment and raw water samples

**Table 7:**
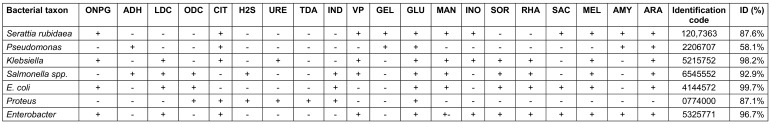
Biochemical characteristics of the isolates based on the API 20E test

**Table 8:**
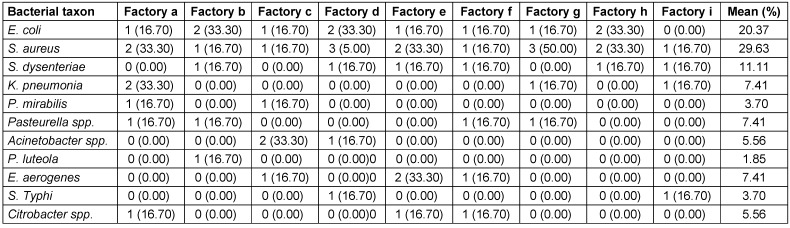
Frequencies of occurrence of the bacterial isolates from nine different brands of sachet water producing factories

**Table 9:**
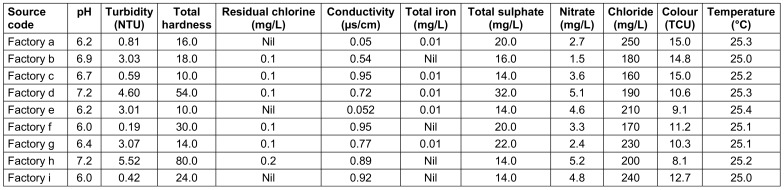
Physicochemical parameters of raw water from the sachet-water factories

**Table 10:**
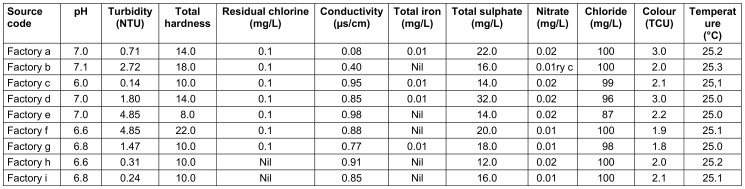
Physicochemical parameters of treated water from the various sachet-water factories
